# Manual therapy regulates oxidative stress in aging rat lumbar intervertebral discs through the SIRT1/FOXO1 pathway

**DOI:** 10.18632/aging.203949

**Published:** 2022-03-15

**Authors:** Chongjie Yao, Guangxin Guo, Ruixin Huang, Cheng Tang, Qingguang Zhu, Yanbin Cheng, Lingjun Kong, Jun Ren, Min Fang

**Affiliations:** 1 School of Acupuncture-Moxibustion and Tuina, Shanghai University of Traditional Chinese Medicine, Shanghai 201203, P.R. China; 2 Yueyang Hospital of Integrated Traditional Chinese and Western Medicine, Shanghai University of Traditional Chinese Medicine, Shanghai 200437, P.R. China; 3 Research Institute of Tuina, Shanghai Academy of Traditional Chinese Medicine, Shanghai 200437, P.R. China; 4 Shanghai Municipal Hospital of Traditional Chinese Medicine, Shanghai University of Traditional Chinese Medicine, Shanghai 200071, P.R. China

**Keywords:** manual therapy, aging, intervertebral disc degeneration, oxidative stress, SIRT1/FOXO1 pathway

## Abstract

With the increasing burden of a globally aging population, low back pain has become one of the most common musculoskeletal disorders, caused mainly by intervertebral disc (IVD) degeneration. There are currently several clinical methods to alleviate back pain, but there is scarce attention paid as to whether they can improve age-related IVD degeneration. It is therefore difficult to conduct an in-depth evaluation of these methods. A large number of clinical studies have shown that manual therapy (MT), a widely used comprehensive alternative method, has effects on pain, the mechanisms of which require further study. In this study, MT was performed on aging rats for 6 months, and their behaviors were compared with those of a non-intervention group of aging and young rats. After the intervention, all rats were examined by X-ray to observe lumbar spine degeneration, and the IVD tissues were dissected for detection, including pathological staining, immunofluorescence, Western bolt, etc. This study demonstrated the possibility that MT intervention delay the lumbar IVD degeneration in aging rats, specifically improving the motor function and regulating senescence-associated β-galactosidase, p53, p21, p16, and telomerase activity to retard the senescence of cells in IVDs. Moreover, MT intervention can modify oxidative stress, increase the expression of SIRT1 and FOXO1 in IVDs and decrease ac-FOXO1 expression, suggesting that MT can reduce oxidative stress through the SIRT1/FOXO1 pathway, thereby playing a role in delaying the aging of IVDs. This study shows that drug-free, non-invasive mechanical interventions could be of major significance in improving the physical function of the elderly.

## INTRODUCTION

Low back pain (LBP) is a common symptom of intervertebral disc (IVD) degeneration, which is one of the most common musculoskeletal disorders and the leading cause of disability [1]. An IVD is composed of fibrocartilaginous tissue and consists of the nucleus pulposus (NP), annulus fibrosus (AF), and cartilage endplate (CEP) [2]. Unlike other tissues in the body, the IVD’s structural integrity begins to show a degenerative state in early stages of human life as the result of physiological aging, a state aggravated with age [3, 4]. Delaying senescence has therefore, been proposed as a feasible therapeutic target for degenerative disc disease [5]. Despite the reports of certain treatments for relieving pain and symptoms [6–8], these methods’ role in delaying IVD aging is rarely explained on the molecular level, which creates challenges to their clinical application.

Given that the accumulation of senescent disc cells is considered an important factor leading to IVD degeneration, the number of studies discussing disc cell senescence has been steadily increasing [9–11]. Various causes for disc cell senescence have been identified, with oxidative stress one of the major contributors [12]. According to the free radical theory of senescence, the decline of tissue and organ function is closely related to the oxidative stress induced by reactive oxide species (ROS), in particular mitochondrial-derived ROS [13]. With the aging process, ROS levels in IVD increase significantly, including those of hydrogen peroxide, hydroxyl radicals, superoxide anions, and nitric oxide, which are byproducts of cellular oxidative metabolism [14]. As an important mediator, ROS can regulate extracellular matrix metabolism, pro-inflammatory factor phenotype, autophagy, and apoptosis, indicating that oxidative stress in the degenerative disc microenvironment can play a crucial role in the pathological development of disc cell senescence [15, 16].

When cells are exposed to oxidative stress over time, they become senescent and lose the ability to proliferate, showing stagnation of the cell cycle in the G0/G1 phase and the activation of senescence-related p53-p21-phosphorylated retinoblastoma (p-Rb) and p16-pRb pathways [17, 18]. Silent mating type information regulation 2 homolog-1 (SIRT1) is a nicotinamide dinucleotide (NAD+)-dependent deacetylase, which protects against cell senescence by regulating p53, p21, and p16, as well as molecules involved in DNA damage and repair [19]. Previous studies have investigated the role of SIRT1 in delaying aging-related diseases and have explored the relationship between SIRT1 and oxidative stress. On one hand, SIRT1 has been shown to regulate cellular oxidative stress through the deacetylase function [20, 21]; on the other hand, oxidative stress can affect SIRT1 activity by regulating gene expression at the transcriptional level and through post-translational modifications [22, 23]. Recent studies have implied that SIRT1 might relieve oxidative stress induced senescence by regulating the SIRT1/forkhead box O (FOXO)1 pathway. Alvarez et al. [24] showed that decreasing FOXO1 expression led to cell senescence and tissue degeneration in IVDs, indicating that FOXO1 is an important regulator for maintaining IVD homeostasis during aging. As the upstream of FOXO1, SIRT1 can reinforce the resistance to oxidative damage of tissue and delay aging by inhibiting FOXO1 acetylation [25]. However, the functions of the SIRT1/FOXO1 pathway in the aging of NP cells (NPC) has not been well studied.

As a widely used complementary and alternative therapy, manual therapy (MT) is performed by the hands on external areas of the body, which can affect the internal physiological state [26]. In China, Tuina is a traditional style of MT that involves pressing, pinching, and kneading the body [27] and has demonstrated positive effects for treating LBP in clinical practice [28–30]. However, there have been few studies on the effects of MT on tissue degeneration beyond pain relief, although an association has been shown between LBP and age-related IVD degeneration [31]. Van et al. [32] reported that MT can synthesize free myofibroblast protein in muscle and can therefore be used as a potential therapeutic option for aged muscle impairment, indicating that MT might alleviate the tissue degeneration caused by aging. In addition, a number of studies [33, 34] have suggested that MT can reduce oxidative stress, which might be one of the mechanisms of the analgesic effect.

However, whether MT can inhibit the onset of age-related IVD degeneration via antioxidation remains unknown. The objective of this study is therefore to explore whether MT can be a useful intervention on the aging of lumbar IVDs by regulating oxidative stress and to determine the role that the SIRT1/FOXO1 pathway plays in the intervention.

## RESULTS

### Effects of MT on the behavior of aging rats

**Figure 1:**
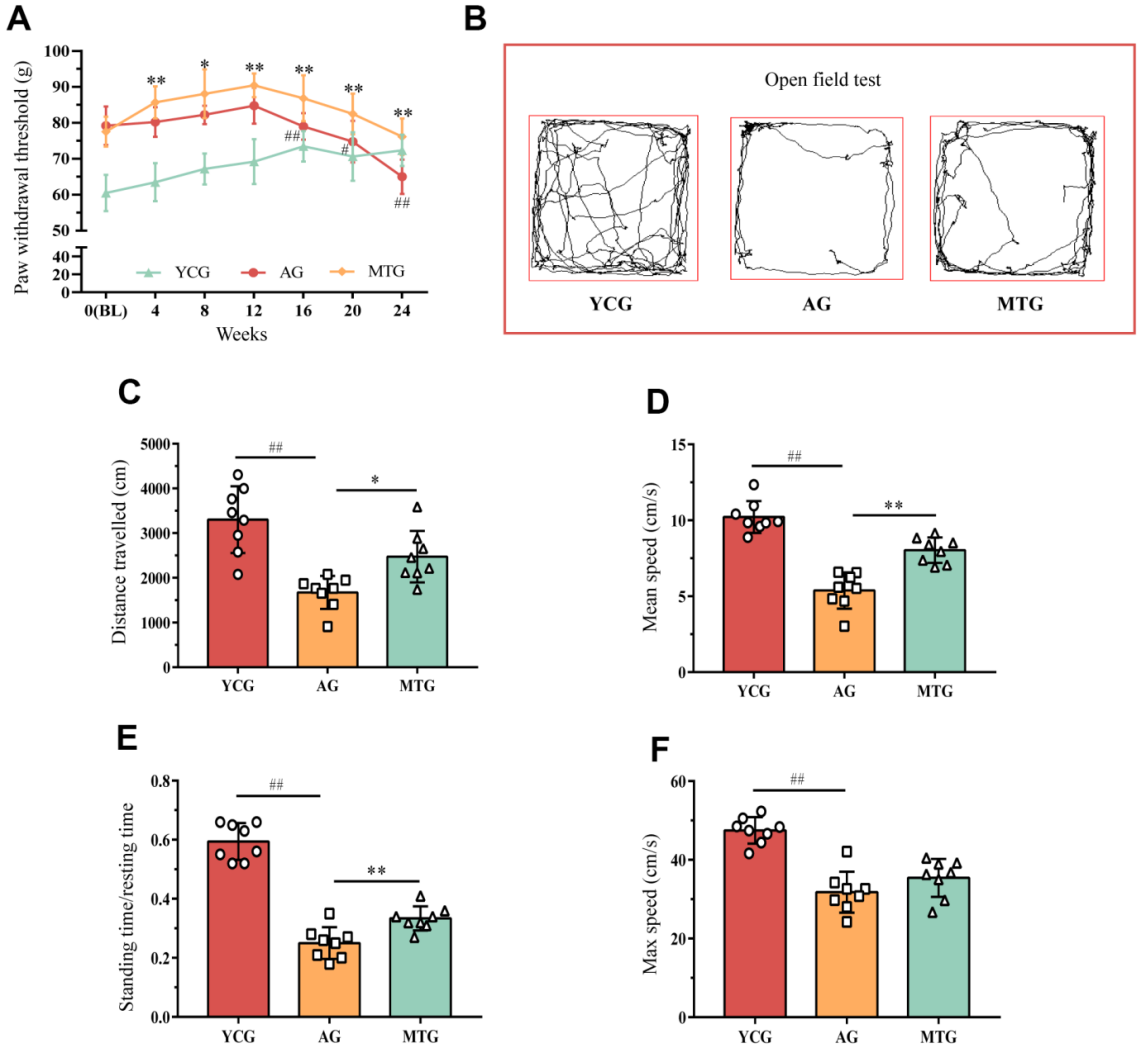
**Effects of MT on the behaviors of aging rats.** (**A**) The PWT of rats were obtained by stimulating to assess their mechanical hyperalgesia. Compared with the previous time point of this group, ^#^*P* < 0.05, ^##^*P* < 0.01; compared with the AG at the same time point, ^*^P < 0.05, ^**^P < 0.01. Rats in the YCG were 6 months old at BL; rats in the AG and MTG were 12 months old at BL. (**B**) The open field test was used to measure mobility and voluntary standing to indirectly reflect whether the rats had LBP. (**C**–**F**) Further quantitative analysis was used for the distance traveled, mean and max speed, and the standing time/ resting time of the rats. Values are expressed as the mean ±SD (N = 8). Compared with the YCG, ^##^*P* < 0.01; compared with the AG, ^*^P < 0.05, ^**^P < 0.01. PWT: paw withdrawal threshold, BL: baseline, YCG: young control group, AG: aging group, MTG: manual therapy group.

Due to rats’ inability to accurately convey their feelings, a single behavioral assay might not precisely capture the presence of LBP in these animals. It is therefore, reasonable to use a combination of stimulation and non-stimulation to evaluate pain. The rats’ paw withdrawal threshold (PWT) can be determined through stimulation to assess their mechanical hyperalgesia. As shown in Figure 1A, the PWT of the young rats increased slowly as they aged from 0 to 6 months, an increase that might be related to the enhancement of muscle strength and adaptation to stimulation. This upward trend remained even in the aging rats; the PWT of the 12-month-old rats continued to increase until 15 months of age. From the age of 16 months, the PWT of the rats in the aging group (AG) was significantly lower than that for the previous month (*P* < 0.01), until it decreased to 65.00 ± 4.78 at the age of 18 months. The PWT of the manual therapy group (MTG) was significantly higher than that of the AG at the same time point after the first month of intervention (*P* < 0.05, *P* < 0.01), and the statistically significant difference remained until the age of 18 months, when the PWT of the MTG remained at 76.17 ± 5.07.

The open field test was used to measure mobility and voluntary standing, which is a non-stimulating method to indirectly reflect whether rats have LBP. As shown in Figure 1B, the 6-month-old rats preferred to explore the surrounding environment and run continuously around the field; while the 18-month-old rats seemed to prefer moving slowly at the edge of the field, especially in the corners. Further quantitative analysis (Figure 1C–1F) showed that during the 5-minute test, the distance traveled, the mean and max speed, and the standing time/resting time of the AG were significantly lower than those of the young group (*P* < 0.01). After 6 months of continuous MT intervention, the distance traveled, the mean speed, and the standing time/resting time of the MTG were significantly higher than those of non-intervened rats of the same age (*P* < 0.05, *P* < 0.01); however, the MT intervention did not seem to improve the maximum speed of the aging rats (*P* > 0.05). These results revealed that the aging rats spent less time standing and exploring, which might be partially improved by the MT intervention.

### MT improved degenerative changes in the lumbar IVDs with aging

**Figure 2:**
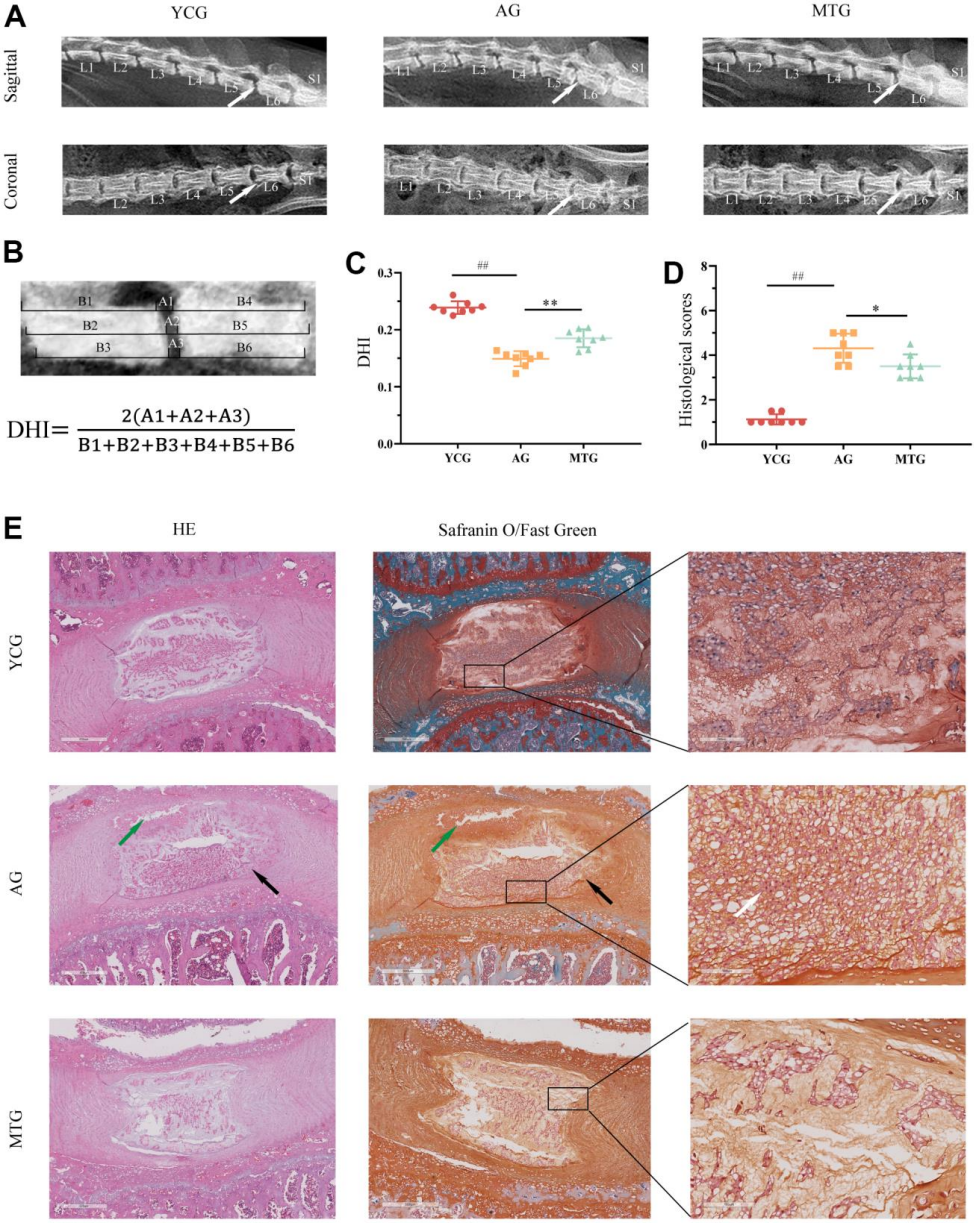
**MT improved degenerative changes in the lumbar IVDs with aging.** (**A**) Radiographs showed the sagittal and coronal view of representative L5/L6 vertebrae. (**B**) DHI was measured on sagittal radiographs with the equation below. (**C**) Quantification analysis was used for DHI. (**D**) Histological scores of disc degeneration were analyzed. (**E**) The tissue sections were stained with HE and Safranin O/Fast Green (bar = 500 and 100 μm). The white arrow indicates severe degenerative phenotypes, the black arrow indicates inner layers buckling inward, and the green arrow shows partial necrosis in the disc. Values are expressed as the mean ± SD (N = 8). Compared with the YCG, ^##^*P* < 0.01; compared with the AG, ^*^P < 0.05, ^**^P < 0.01. DHI: disc height index, YCG: young control group, AG: aging group, MTG: manual therapy group.

To intuitively evaluate the age-related changes in the lumbar IVD, we took radiographs of the rats’ lumbar spine in the coronal and sagittal planes (Figure 2A). The X-ray images indicated that the IVDs of the AG were characterized by height loss and blurring of the CEP boundary, which can be quantified by calculating the disc height index (DHI) (Figure 2B). Compared with the 6-month-old rat lumbar IVDs, the 18-month-old lumbar IVDs showed a significant reduction in DHI (*P* < 0.01), indicating that MT can significantly increase the DHI of aging rats (*P* < 0.01) (Figure 2C).

To observe the degenerative disc phenotypes, the tissue sections were stained with hematoxylin and eosin and Safranin O/Fast Green. The results showed that the young control group (YCG) had healthy discs (Figure 2E), characterized by normal cellularity with a mix of large, vacuolated (notochordal) cells in the NP, and clear demarcation between NP and AF tissues with an intact and tightly arranged structure. On the other hand, the discs of the AG revealed severe degenerative phenotypes, including cell arrangement disorders, pronounced cell loss, and the presence of non-vacuolated cells (Figure 2E, white arrow). The clear distinction between the NP and AF tissue compartments was lost, and some of the inner AF layers showed inward buckling (black arrow). Tissue demarcation between the NP and CEP was indistinct and showed partial necrosis (green arrow). Similarly, the junction between the AF and NP was blurred in the discs of the MTG due to aging. Compared with the AG, however, the rats that underwent MT had improved degenerative disc phenotypes, including cell loss, altered cell distribution and morphology, thus maintaining IVD function. The histological scores revealed (Figure 2D) that the IVD scores of the AG were significantly higher than those of the YCG (*P* < 0.01), while the scores for the MTG were significantly lower than those of the AG (*P* < 0.05).

Taken together, these data suggest that aging rats develop degenerative IVDs, resulting in structural destruction and cellular disorder, and that MT can alleviate the age-related changes in IVDs.

### MT altered the redox balance of lumbar IVDs by promoting the antioxidant capacity

**Figure 3:**
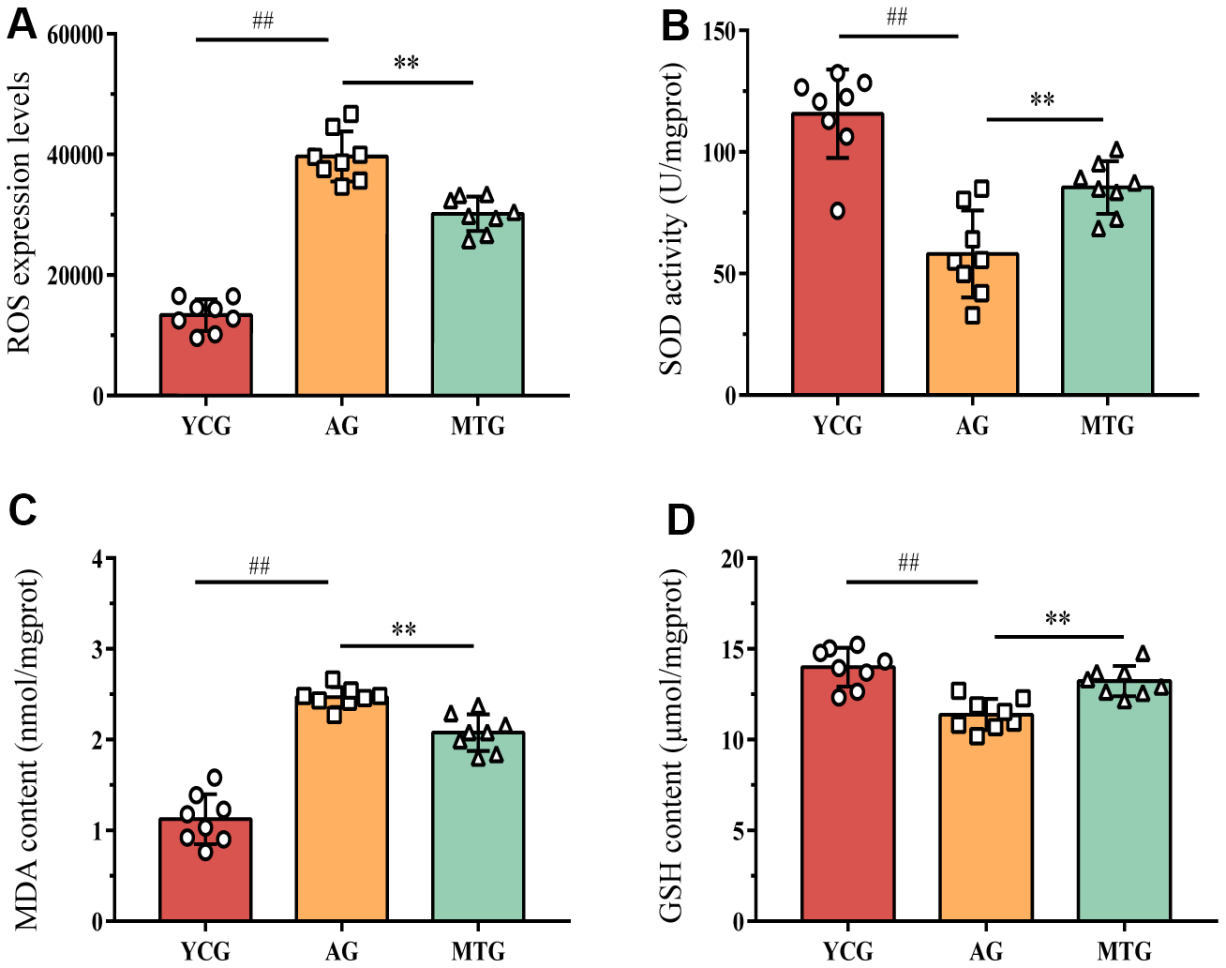
**MT modified the redox balance of lumbar IVDs by promoting antioxidant capacity.** (**A**–**D**) The level of ROS, the activity of superoxide dismutase SOD, and the content of MDA and GSH were detected to assess the effects of MT on oxidative balance. Values are expressed as the mean ± SD (N = 8). Compared with the YCG, ^##^*P* < 0.01; compared with the AG, ^**^P < 0.01. ROS: reactive oxide species, SOD: superoxide dismutase, MDA: malondialdehyde, GSH: glutathione, YCG: young control group, AG: aging group, MTG: manual therapy group.

To assess the effects of MT on the oxidative balance of IVDs, we measured ROS levels, superoxide dismutase (SOD) activity, and the malondialdehyde (MDA) and glutathione (GSH) content. The ROS expression levels in the NP tissues of the aging rats were significantly higher than those of the young rats (*P* < 0.01), suggesting that aging can cause more oxidative stress damage to tissues (Figure 3). As the product of lipid peroxidation, the MDA content of the aging rats was also significantly higher than that of the young rats (*P* < 0.01). In contrast, SOD and GSH are important antioxidant enzymes for countering the overproduction of active free radicals. However, the SOD activity of the NP in the AG was significantly lower than (almost half) that in the YCG (58.06 ± 17.86 vs. 115.71 ± 18.20; *P* < 0.01). Although the GSH content did not decrease substantially (11.38 ± 0.86 vs. 13.99 ± 1.07), the difference was statistically significant (*P* < 0.01). The results indicated that aging decreased IVD antioxidant capacity, disrupting the original redox balance.

The lack of antioxidant capacity and redox imbalance in the lumbar IVDs might be modified by the MT intervention. Compared with the non-intervened aging rats, the rats that underwent MT had enhanced SOD activity and GSH content and decreased ROS and MDA content, differences that were statistically significant (*P* < 0.01).

### MT delayed NPC senescence in lumbar IVDs

**Figure 4:**
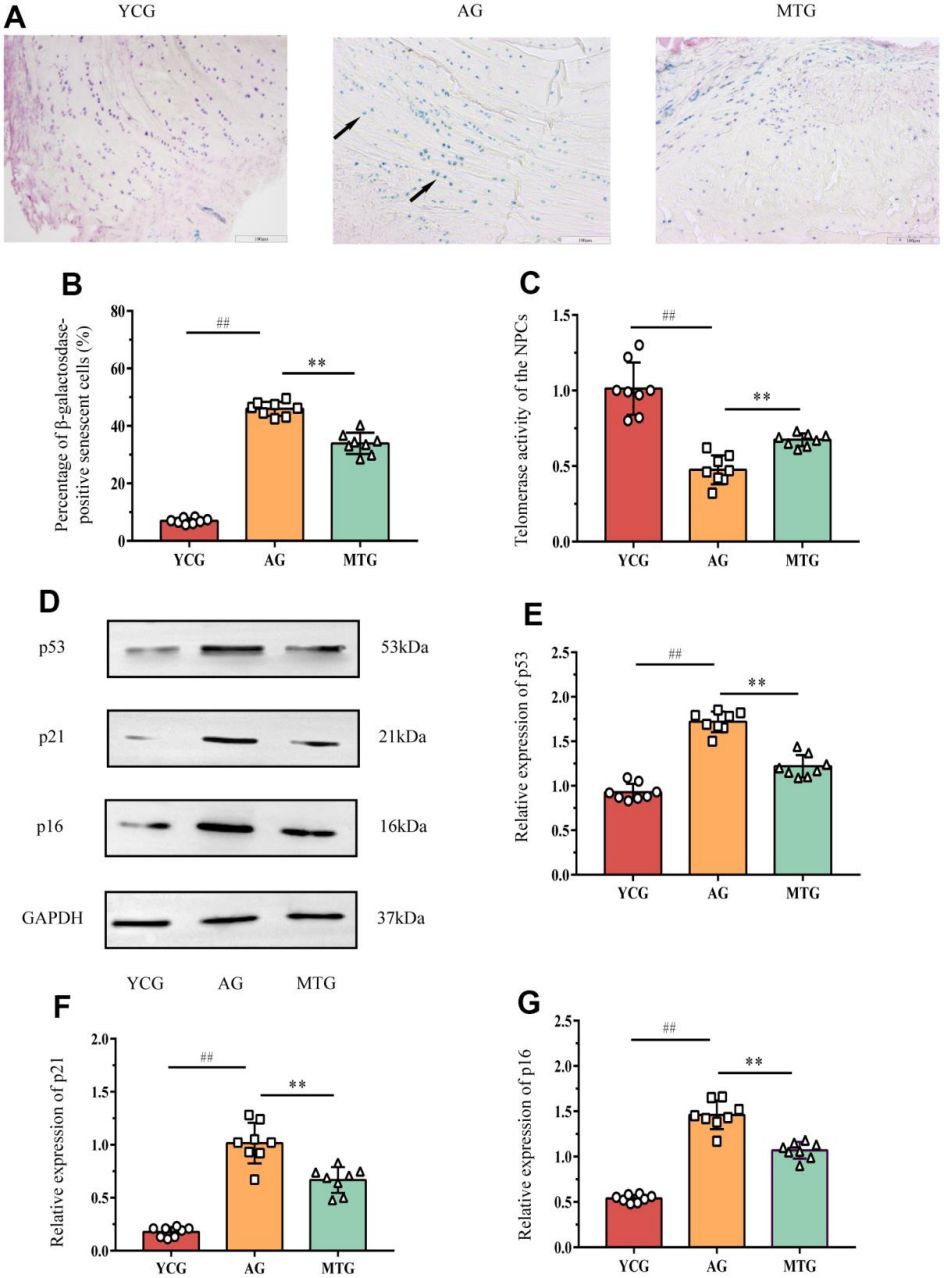
**MT retarded the senescence of NPCs in lumbar IVDs.** (**A**) SA-β-Gal staining showed the cellular senescence in discs (bar = 100μm). The blue cells indicated by the black arrow are senescence-related positive cells. (**B**) The percentage of SA-β-Gal positive cells was calculated. (**C**) Telomerase activity of the NPCs was indirectly reflected by the relative mRNA expression of telomerase reverse transcriptase detected by PCR. (**D**–**G**) Western blot analysis showed the relative expressions of senescence-related p53, p21 and p16 proteins in NPCs. Values are expressed as the mean ± SD (N = 8). Compared with the YCG, ^##^*P* < 0.01; compared with the AG, ^**^ *P* < 0.01. NPC: nucleus pulposus cell, SA-β-Gal: senescence-associated β-galactosidase, YCG: young control group, AG: aging group, MTG: manual therapy group.

To objectively evaluate the effects of MT on aging rat lumbar IVD tissue, we measured the cellular senescence-related indexes. Senescence-associated β-galactosidase (SA-β-Gal) staining showed that the blue positive cells (Figure 4B, black arrow) were densely arranged in the IVD tissues of the aging rats, compared with few positive cells in the young rats. Further statistical analysis (Figure 4B) revealed that the SA-β-Gal-positive cell rate in the IVD tissues of the young rats was significantly lower than that of the aging rats (*P* < 0.01). Moreover, NPC telomerase activity was indirectly reflected by the relative mRNA expression of telomerase reverse transcriptase detected by polymerase chain reaction (PCR). The results (Figure 4C) showed that the NPC telomerase activity in the AG was significantly lower than that in the YCG (*P* < 0.01). These two indicators can be used as relatively specific cell sensitivity markers. Using these markers, we found that MT can significantly reduce the SA-β-Gal-positive cell rate and improve telomerase activity (*P* < 0.01), which suggests that MT can play an important role in delaying cell aging.

The western blot analysis (Figure 4D–4G) showed that the expression of senescence-related p53, p21, and p16 proteins in the NPCs of the AG were significantly higher than those of the YCG (*P* < 0.01) and that MT can significantly lower the expression of these proteins when compared with the non-intervened aging rats (*P*<0.01).

Taken together, these data suggest that aging induces a decrease in NPC telomerase activity in the rat lumbar IVDs, which can activate p53, p21, and p16 proteins regulating cell senescence, thereby accelerating the aging process. MT intervention can inhibit the reduction of telomerase activity, reducing the expression of senescence-related proteins, thereby delaying NPC senescence in lumbar IVDs.

### MT exerted its effects by regulating the SIRT1/FOXO1 pathway

**Figure 5:**
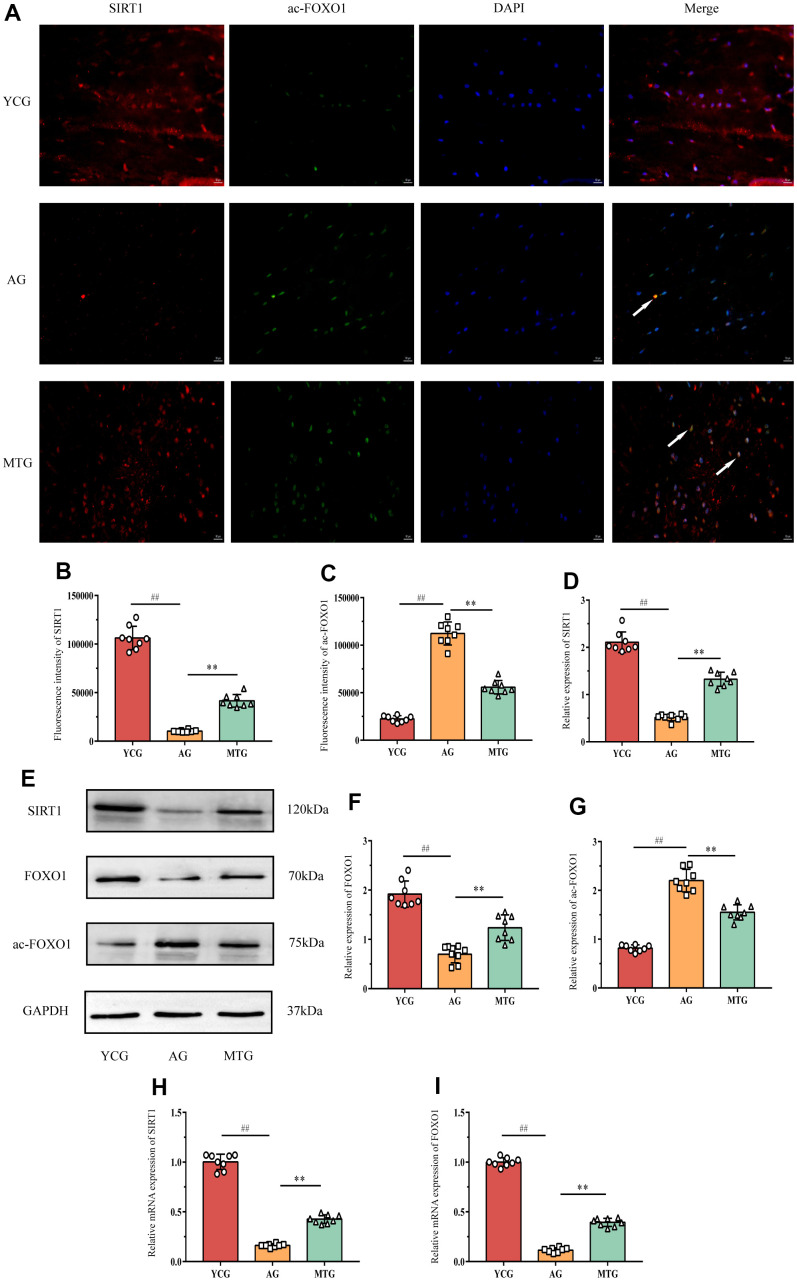
**MT exerted its effects through regulating the SIRT1/FOXO1 pathway.** (**A**) Immunofluorescence staining showed the expressions of SIRT1 (red fluorescent) and ac-FOXO1 (green fluorescent) (bar = 50 μm). The white arrow indicates that the two proteins were co-expressed in the NPCs. (**B,** **C**) The fluorescence intensity of SIRT1 and ac-FOXO1 was analyzed. (**D**–**G**) A Western blot analysis showed the relative expressions of SIRT1, FOXO1 and ac-FOXO1 proteins in NPCs. (**H,** **I**) PCR results showed the relative mRNA expressions of SIRT1 and ac-FOXO1. Values are expressed as the mean ± SD (N = 8). Compared with the YCG, ^##^*P* < 0.01; compared with the AG, ^**^P < 0.01. SIRT1: silent mating type information regulation 2 homolog-1, FOXO1: forkhead box O1, YCG: young control group, AG: aging group, MTG: manual therapy group.

To investigate the possible mechanism by which MT reduces lumbar IVD degeneration, we analyzed the changes in the SIRT1/FOXO1 pathway in the NPCs. Immunofluorescence staining showed that SIRT1 (Figure 5A, red fluorescent) and ac-FOXO1 (Figure 5A, green fluorescent) were co-expressed in the NPCs of the MTG (Figure 5A, white arrow). However, ac-FOXO1 was difficult to observe in the NPCs of the YCG, and there was a large amount of SIRT1. In contrast, there was only a small amount of SIRT1 in the AG, and ac-FOXO1 was mainly expressed in the NPCs. These results suggest that SIRT1, which is abundantly expressed in the NPCs of the young rats, can inhibit FOXO1 acetylation to maintain NPC homeostasis; the lack of SIRT1 in the aging rats can increase the degree of FOXO1 acetylation and disrupt the original homeostasis. The MT intervention can significantly increase SIRT1 expression in the NPCs and reduce ac-FOXO1 expression (*P* < 0.01), thereby reducing the effects of NPC aging (Figure 5B, 5C).

The western blot analysis (Figure 5E) also showed changes in the SIRT1, FOXO, and ac-FOXO1 proteins in the pathway. The results for SIRT1 and ac-FOXO1 were consistent with those of the immunofluorescence (Figure 5D, 5G). Compared with the YCG, the AG had significantly decreased FOXO1 expression in the NPCs (*P* < 0.01), which might have been caused by acetylation of a large amount of FOXO1, and MT might indirectly increase FOXO1 expression by inhibiting acetylation (*P* < 0.01). In addition, the PCR results for SIRT1 and FOXO1 further confirmed the above results (Figure 5H, 5I). Thus, MT might exert its effects by regulating the SIRT1/FOXO1 pathway.

## DISCUSSION

IVD degeneration is a common risk factor for LBP in the elderly, which is a characteristic of aging [31]. Human lumbar IVDs undergo age-related pathological changes, including collapse, herniation, and tears, which can be significant causes of related symptoms [35]. In particular, patients with LBP have shorter lumbar IVDs than those without LBP, which is particularly common in the lumbosacral level L5/S1, followed by L4/L5, which suggests that the vulnerability of these levels in humans might be partly due to the vertical pressure exerted by their upright posture [36]. From this point of view, finding a suitable animal model appears difficult. Most studies [37–39] on disc degeneration have therefore used surgically created animal models to cause similar changes. However, IVD degeneration also reveals that age-related changes in IVD structure might be more directly reflected in quadrupeds. We therefore, chose aging rats as the research subjects in this experiment. Despite the rats’ quadrupedal stance, they often need to stand upright to eat and explore their external environment, which shows that rat lumbar IVDs might have similar mechanical properties to those of humans.

With the growing problem of the aging global population, MT has become an effective means to improve the health of the elderly and even act as a developmental strategic plan in a number of countries [40]. However, there are few studies on the effects of MT on age-related diseases. Previous studies have suggested that MT can improve the age-related symptoms of postmenopause [41], Parkinson’s disease [42], and dementia [43], and the effects are more obvious with long-term interventions. These effects by MT are thought to be achieved through the stimulation of baroreceptors on the body surface, transmitting signals to the vagus nerve, so as to inhibit the function of the hypothalamic-pituitary-adrenocortical axis [44], which is also a vital method for delaying aging [45]. However, we still have no direct evidence of the effectiveness of MT, which makes its promotion difficult due to the lack of evidence.

In this study, we observed the changes in the mechanical stimulation threshold and spontaneous behavior of rats of different ages. The rats exhibited behavioral changes related to IVD degeneration with increasing age, results that were partially similar to those of a previous study [31]. The rats’ sensitivity to mechanical stimulation was evaluated by measuring their PWT on a monthly basis. The PWT of the non-intervened rats showed an upward trend from 0 to 6 months and from 12 to 15 months, and decreased significantly compared with the previous month from the age of 15 months, indicating that their PWT began to decrease. We also compared the changes in spontaneous behavior between the 18-month-old and 6-month-old rats and found that the young rats had good motor ability and often stood on their hind limbs to free their front paws for exploring. The aged rats kept moving and exploring novel environments but opted to remain on all four limbs on the ground. According to a previous report [31], the aged rats’ reluctance to stand is unlikely to be due to muscle atrophy or loss of strength because muscle mass increases with age and remains proportional to body weight. Instead, the reluctance might be related to discomfort due to LBP or other musculoskeletal problems when standing. Taking into consideration the exact efficacy of MT in alleviating LBP [7, 46], we observed the effects of MT on the aging of rat IVDs by pressing and kneading the Shenshu (BL23) points. We selected BL23 points as the MT site because they are located on both sides of the spine, so mechanical loading can directly relax the lumbar muscles, and they have been reported to delay the effects of aging [47–49]. Through behavioral testing, we found that the pain tolerance and behavior style of the aged rats undergoing the MT intervention were significantly improved. However, we were unable to determine whether MT alone alleviated LPB or simultaneously relieved lumbar IVD degeneration; we therefore conducted the following study.

A longitudinal study of the elderly revealed the association between LBP and loss of IVD height [50]. In our study, radiographs showed the sagittal and coronal views of representative L5/L6 vertebrae, indicating loss of IVD height, increased IVD width, and reduced DHI, which emerge during old age in rats, in a similar manner as in humans [51]. We also observed histological changes at 18 months in the rats’ lumbar IVDs. As the IVD ages, severe degenerative structural defects such as cell arrangement disorders, pronounced cell loss, and the presence of non-vacuolated cells become more common, indicating that progressive degenerative changes occur spontaneously with advanced age in rats despite the absence of injury and lifestyle-related risk factors that cause IVD degeneration in humans. Our results support the effects of MT on the structural improvement of rat lumbar IVDs, indicating that MT can not only alleviate LBP but also delay IVD degeneration.

Senescence in IVD cells, especially NPCs, has recently emerged as a major driver of disc aging, leading to changes in IVD structure and function [52]. SA-β-gal is a relatively specific cell senescence marker that reflects increased expression of lysosomal β-galactosidase protein in senescent cells [53]. Compared with undegenerated IVDs, degenerated discs show an increase in SA-β-gal-positive cells [54]. Moreover, telomerase activity is another marker for evaluating cellular senescence [55], because decreased telomerase activity can activate the expression of tumor suppressor gene p53 and p16 by mimicking DNA damage signals, which in turn reduces the degree of pRB protein through the p53-p21-pRB and p16-pRB pathways, eventually inducing cellular senescence. In our study, the increase in SA-β-gal-positive cells and senescence-related p53, p21, and p16 protein expression and the decrease in telomerase activity in the NPs of the aged rats suggest cellular senescence in degenerative IVDs. We found that the degree of IVD aging in the aged rats that underwent 6 months of MT was lower than that of the rats that did not undergo the intervention, and the expression of the senescence-related proteins was also significantly reduced, suggesting that MT delayed NPC senescence in the lumbar IVDs. Oxidative stress induced by various risk factors has been considered closely associated with NPC senescence, and excessive oxidative stress results in accelerated aging and IVD degeneration [56]. Our study verified the results of previous studies [2, 57] and showed the antioxidant capacity and redox imbalance in aged discs, which was specifically manifested in the large increase in ROS and decrease in antioxidant enzymes, resulting in the accumulation of peroxide products. Our study confirmed that MT can modify the redox balance of lumbar IVDs by promoting the antioxidant capacity, which might be one of the important mechanisms of MT in delaying IVD degeneration.

A previous study [24] observed a reduction in FOXO1 expression in mouse lumbar IVD in early aging, which compromised the ability of IVD cells to neutralize ROS, suggesting that FOXO1 expression and activity might have an impact on disc cell homeostasis by decreasing oxidative damage. However, the mechanism by which FOXO1 expression is reduced in aging IVD has not been studied. Taking into consideration the SIRT1 function in mitigating senescence-related dysfunction in NPCs [58], we assumed that FOXO1 might be regulated through the SIRT1/FOXO1 pathway. To confirm our hypothesis, we studied the changes in SIRT1, FOXO1, and ac-FOXO1 in the pathway. Immunofluorescence staining showed that SIRT1 was abundantly expressed in young NPCs, while ac-FOXO1 was difficult to find, which was in contrast to the expression trend in aged NPCs. The results of the western blot and PCR were consistent with the above trends. In the NPCs of the aged rats with low SIRT1 levels, FOXO1 expression was also decreased, while ac-FOXO1 expression was increased. Through the MT intervention, SIRT1 levels in the NPCs of the aged rats increased significantly, suggesting that the decrease in ac-FOXO1 might be due to SIRT1 deacetylation, which indirectly increased FOXO1 expression by promoting the nuclear translocation and activation of FOXO1.

There are a number of limitations that should be noted in this study. First, due to the short project time, the overall changes in the rats from 0 to 18 months were not be observed; instead, we compared the aged and young rats, which might have a certain effect on the results (especially the behavior tests). Second, the quantitative standard of the MT intervention referred to our previous study on LBP, and further study is needed to determine whether there was a more suitable standard for this research. While the present findings demonstrated that MT might alleviate oxidative stress through the SIRT1/FOXO1 pathway and delay IVD degeneration caused by aging, a causal relationship needed to be established. Thus, we need to improve the experimental design in future studies.

Nevertheless, our study confirmed the view that MT might be beneficial for patients with age-associated diseases, for which immunosuppressants are often ineffective; and physical therapy should be recommended as an alternative [59]. With the problem of the global aging population becoming increasingly serious, it is of major importance to develop external mechanical interventions to improve the physiological function of the elderly in a drug-free, non-invasive manner.

In conclusion, this study demonstrated the possibility that an MT intervention can delay lumbar IVD degeneration in aged rats, specifically improving the motor function and delaying NPC senescence. Moreover, the MT intervention can regulate oxidative stress, increase SIRT1 and FOXO1 expression in NPCs and decrease ac-FOXO1 expression, which suggests that MT can reduce oxidative stress through the SIRT1/FOXO1 pathway, thereby playing a role in delaying IVD aging.

## MATERIALS AND METHODS

### Animals

Eight young male Sprague–Dawley rats (2 weeks) and 16 adult males (12 months, weight 700 ± 50 g) were obtained from Shanghai Jihui Laboratory Animal Co., Ltd. [certificate number: SCXK (Shanghai)2017-0012] and housed in the Laboratory Animal Center of Yueyang Hospital of Integrated Traditional Chinese and Western Medicine. All the rats were fed in standard laboratory conditions (22~27° C, 50%–70% indoor humidity) under a 12-h light-dark cycle with rat chow and water ad libitum for one week before the experiment. To alleviate pain and avoid injury, the animal feeding, caring, and intervention were conducted in accordance with procedures approved by the Experimental Animal Ethics Committee of Yueyang Hospital of Integrated Traditional Chinese and Western Medicine (ethics number: YYLAC-2020-095).

### Grouping and intervention

**Figure 6:**
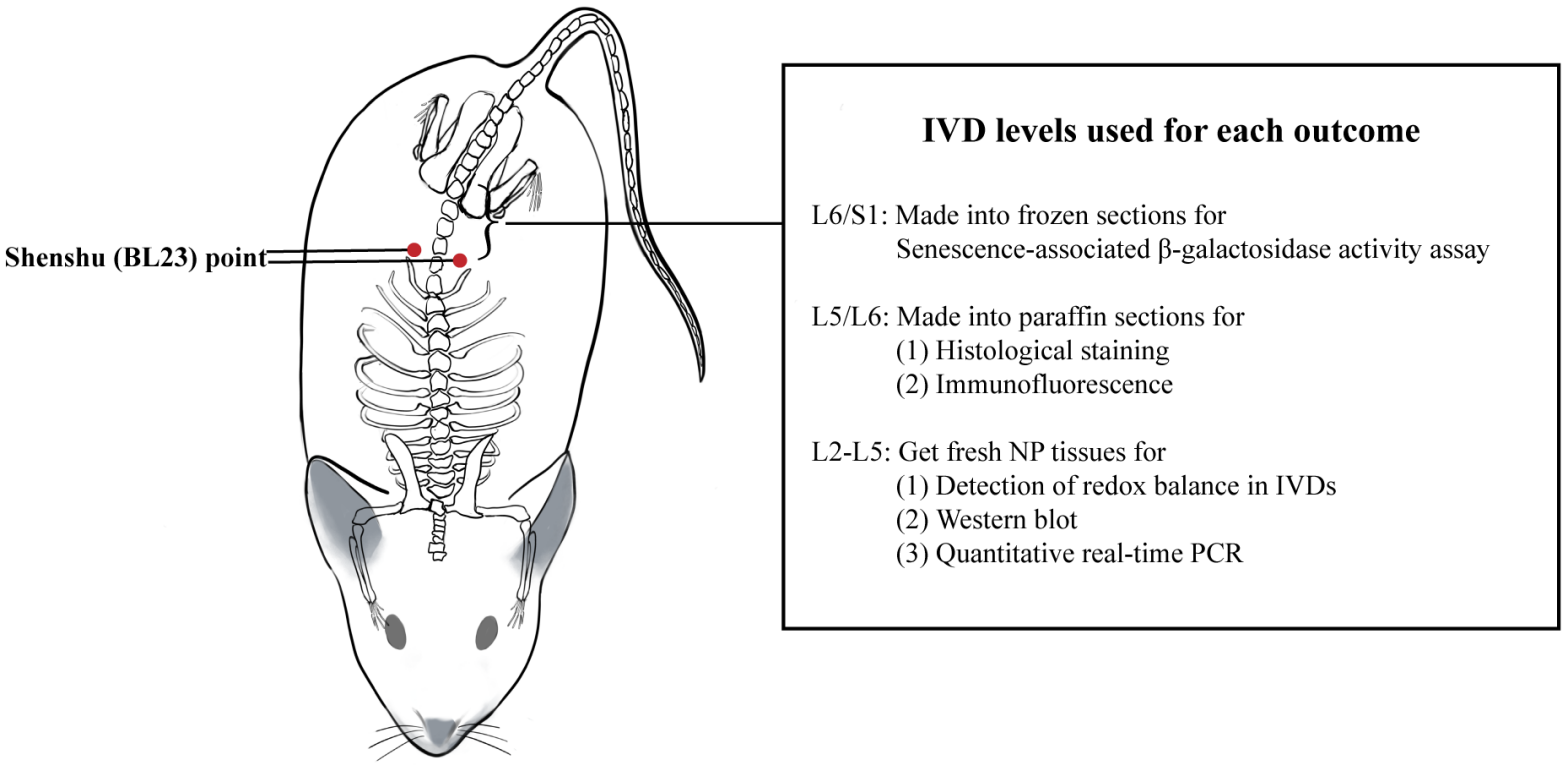
**Location of Shenshu (BL23) point and IVD levels used for each outcome.** IVD: intervertebral disc.

The 16 adult male Sprague-Dawley rats were randomly divided into the aged group (AG) and the manual therapy group (MTG) (N = 8). The 8 immature male rats comprised the young control group (YCG). The MTG underwent manual therapy on the acupoint beside the spine from 12 months of age. The specific operation was as follows: 1) before the intervention, the rats were placed in the experimenters’ hands 30 min prior to the operation to adapt; (2) the right thumb was used to press and knead the Shenshu (BL23) points on the back of the rat, which are located on both sides of the second lumbar vertebrae and on the erector spinalis muscle (Figure 6); (3) to maintain the amount of stimulation during the manipulation, the right thumb was operated with a wireless tactile measurement finger sleeve (FingerTPS, Pressure Profile System, USA), with a pressure of 5 N and a frequency of 2 Hz [27]; 4) the rats underwent the intervention alternately on the left or right side of the spinal column, one acupoint each time for 10 min, once a day, 6 days a week for 6 months; and 5) after the MT intervention, the rats were pacified before being put into the cage. The AG and YCG underwent no intervention but were grabbed and fixed in the same way as the MTG.

### Behavioral testing

Behavioral tests were conducted for 6 months during the light cycle between 9 am and 5 pm. The rats were habituated to the experimental environment for 2 h daily 3 days before testing. The mechanical hyperalgesia test was performed on the rats every month, once a day for 3 consecutive days, and the mean results of the 3 tests were taken as the final behavioral test results of the month. The open field test was performed after the 6-month intervention, repeated tests and data processing method was the same as above. The tests were implemented, recorded and supervised by 3 experimenters, respectively, and rotated after each test to reduce the experimental error as much as possible.

An electric von Frey aesthesiometer (IITC/Life Science, USA) was applied to test the mechanical hyperalgesia of the rats, as previously reported [60]. The tip head was aimed at the central part of the left hind foot of the rat and was raised at a constant speed for stimulation. When the aesthesiometer recorded the maximum amount of stimulation making the rat withdraw, and the value of this force was assigned to the paw withdrawal threshold (PWT). To prevent sensory sensitization caused by frequent stimulation, the right foot should be stimulated at least 20 s after the measurement of the left foot. The left and right feet of each rat were repeatedly measured 3 times. The maximum and minimum of the 6 values were deleted, and the mean of the remaining 4 values was deemed as the rat’s PWT in one test [61].

The open field test consisted of a black square field (100×100 cm) surrounded by 30-cm high walls made of translucent plastic, which was placed in a quiet and well-lit room. The test was initiated by placing a single rat in the center of the field and allowing it move freely for 5 min while being photographed by a camera. The field was cleaned with low alcohol after every test, and the next test was carried out after the alcohol evaporated completely. The behaviors of the rats were measured by the experimenter and by analysis software (SMART v3.0); the main parameters were as follows: (1) distance traveled: the total travel distance of the rats in the field; (2) mean speed and max speed: the average speed of the rats in the field and the maximum speed at a certain time point; (3) resting time: the time during which the rats did not move horizontally (including standing time); (4) standing time: the time of rats standing on their hind legs (including wall climbing).

### Radiography and relative height of disc measurement

After the final behavioral testing, images of the lumbar spine were captured with X-ray imaging and irradiation systems (Samsung XGEO, South Korea). Digital images were obtained at 55 kVp under identical imaging conditions using identical acquisition parameters. The rats were anesthetized with 2% pentobarbital sodium and placed in the X-ray chamber on their ventral side with all limbs extended away from the midline in a natural position to obtain coronal images. The rats were then placed on their side in a natural position with the tail extended to obtain sagittal images. Disc height indices (DHI) were measured using ImagePro plus 6.0, by averaging measurements from the anterior, middle, and posterior portions of the IVD (L5/L6) from a sagittal plane and dividing it by the average measurements of the anterior, middle, and posterior portions of the adjacent vertebral bones [62].

### Histological staining

After 6 months of intervention, all rats were euthanized. Intact lumbar spinal segments and IVD tissues from L5/L6 were collected and fixed in 4% formaldehyde for 48h, decalcified in 10% ethylenediaminetetraacetic acid (EDTA) for 1 month. Tissues were then dehydrated in gradient alcohol and embedded in paraffin. 4 μm sections were cut and stained with either hematoxylin and eosin (HE) or Safranin O/Fast Green according to standard procedures. Images were acquired using a microscope (Leica, Germany) and were scored by 2 independent observers using the modified Thompson grading scale [63] to evaluate the degeneration of IVDs.

### Detection of redox balance in IVDs

Fresh NP tissues dissected from L2-L5 were ground and homogenized then lysed thoroughly in RIPA Lysis Buffer (Beyotime, China) at a concentration of 10ul/mg. The cell lysate was centrifuged at 12,000×g for 5 min, and the concentration of the supernatant was calculated by using a BCA protein concentration assay kit (Beyotime, China). Based on the concentration, the tissue homogenate was diluted to 1 mg/ml with PBS.

Test kits (Nanjing Jiancheng, China) were used to detect the level of ROS, the activity of SOD, and the content of MDA and GSH according to the instructions. Additionally, 2, 7- dichlorofluorescein diacetate (DCFH-DA) was used as a probe for ROS, and the fluorescence intensity of ROS was measured at 488nm/525nm with a microplate reader (Thermo Scientific, USA) to determine the level of ROS.

### Senescence-associated β-galactosidase activity assay

Fresh IVDs were dissected from L6/S1, directly embedded in optimal cutting temperature compound, and stored at −80° C to detect the activity of SA-β-Gal in IVDs. Next, 5 μm sections were cut and stained with SA-β-Gal kit (Beyotime, China) according to the manufacturer’s protocol. Before mounting them with resin, sections were stained again with eosin for 10 sec. The number of cells positive (blue) for localization of SA-β-Gal and total cells (blue and red) in the field were counted using the ImagePro plus 6.0 under 400× magnification. The ratio was recorded as the percentage of positive cells. Three fields were randomly selected from each section to calculate the average percentage of SA-β-Gal positive senescent cells.

### Immunofluorescence

After deparaffinization and rehydration, the prepared paraffin sections were treated with citric acid buffer (0.01 mol/L, pH6.0) at 98° C for 15 min for antigen retrieval and were cooled to room temperature for 1 h, followed by incubation with 1% hydrogen peroxide solution for 10 min to block endogenous peroxidase activity. The sections were then incubated in 5% bovine serum albumin (BSA) (Beyotime, China) at room temperature for 30 min, followed by primary antibody incubation (SIRT1, 1:50, CST, 8469s; ac-FOXO1, 1:50, Affinity Biosciences, AF2305) overnight at 4° C. After 12 h, the sections were rewarmed in an oven at 37° C for 45 min and incubated with fluorescein-conjugated secondary antibodies (1:100, Sanggon Biotech, China) at room temperature for 30 min in the dark. Nuclei were stained with 4,6-diamidino-2-phenylindole (DAPI) (Beyotime, China) for 2 min. Three fields were randomly selected from each section to calculate the average fluorescence intensity of the target protein under 400× magnification.

### Western blot

The extraction protocol for total protein quantification of fresh NP tissues was described above. After adjusting the protein concentration, 20 μg tissue lysates were separated by 12% SDS-PAGE gel using electrophoresis and transferred to PVDF membranes. The membranes were incubated with primary antibodies (p53, 1:1000, Abcam, ab26; p21, 1:1000, Abcam, ab109520; p16, 1:1000, Abcam, ab51243; p53, 1:1000, Abcam, ab26; SIRT1, 1:1000, CST, 8469s; FOXO1, 1:1000, CST, 2880s; ac-FOXO1, 1:1000, Affinity Biosciences, AF2305; GAPDH, 1:5000, CST, 5174s) overnight at 4 after blocked by 5% BSA at room temperature for 2 h. The membranes were rinsed 3 times with TBST and incubated with horseradish peroxidase (HRP)-labeled secondary antibody (1:5000, Sanggon Biotech, China) at room temperature for 2 h. The final results were acquired using the ChemiDoc MP system (Bio-Rad, USA) with enhanced chemiluminescence (ECL) kit (Millipore, USA) and were quantified using the ImagePro plus 6.0.

### Quantitative real-time PCR

Reverse transcription was performed with a PrimeScript RT reagent Kit with gDNA Eraser (TaKaRa Bio, Japan). Real-time polymerase chain reaction (RT-PCR) was performed on a PCR System (Bio-Rad, USA) with SYBR Premix Ex Taq (TaKaRa, Japan). The reaction conditions were a single cycle of 95° C for 5 min, and 40 cycles of 95° C for 15 sec, 60° C for 30 sec. GAPDH was used as a reference, each sample was tested in triplicate. The relative expression of the target gene was calculated using the 2^-ΔΔCt^ method. The primers used in the study are shown in Table 1.

**Table 1:** Primers used for RT-PCR analysis.

**Gene**	**Forward (5′-3′)**	**Reverse (5′-3′)**
GAPDH	AGGTGACCGCATCTTCTTGT	TACGGCCAAATCCGTTCACA
FOXO1	CCCAATCTCGGAGCGACAC	GCAGGCTCAGGTTGCTCATA
SIRT1	ATCTCCCAGATCCTCAAGCCA	CTTCCACTGCACAGGCACAT
TERT	TTCCTTCCACCAGGTGTCATC	AGCCAGCACATTCCTCTCAC

### Statistical analysis

SPSS 21.0 software was used for the statistical analysis. Normality and homogeneity of variance tests were performed on the data. The data are presented as mean ± standard deviation (SD) if they were normally distributed; data that did not conform to the normal distribution are presented as the median (*P*25 and *P*75). If the data conformed to the normal distribution, the comparison of differences among groups was performed using one-way ANOVA. If the variances were homogeneous, a pairwise comparison was performed using the least significant difference test; if the variances were not homogenous, Dunnett’s T3 test was used for pairwise comparison. If the data did not conform to the normal distribution, a nonparametric Kruskal-Wallis H test were performed and using the Nemenyi method for pairwise comparison. Repeated measurement analysis of variance was used to analyze the data on the same observation index at various time points between groups. If the results were statistically different, a multivariate analysis of variance was used for pairwise comparison. The significance level of the statistical examination was α = 0.05. Therefore, *P* <0.05 indicated a significant difference.
